# DMSO increases efficiency of genome editing at two non-coding loci

**DOI:** 10.1371/journal.pone.0198637

**Published:** 2018-06-04

**Authors:** George Stratigopoulos, Maria Caterina De Rosa, Charles A. LeDuc, Rudolph L. Leibel, Claudia A. Doege

**Affiliations:** 1 Department of Pediatrics, Columbia University, New York, NY, United States of America; 2 Naomi Berrie Diabetes Center, Columbia University Medical Center, New York, NY, United States of America; 3 Columbia Stem Cell Initiative, Columbia University Medical Center, New York, NY, United States of America; 4 New York Obesity Nutrition Research Center, Columbia University, New York, NY, United States of America; 5 Institute of Human Nutrition, Columbia University, New York, NY, United States of America; 6 Department of Pathology and Cell Biology, Columbia University, New York, NY, United States of America; Utah State University, UNITED STATES

## Abstract

Clustered Regularly Interspaced Short Palindromic Repeats (CRISPR)/CRISPR-associated protein-9 (Cas9) has become the tool of choice for genome editing. Despite the fact that it has evolved as a highly efficient means to edit/replace coding sequence, CRISPR/Cas9 efficiency for “clean” editing of non-coding DNA remains low. We set out to introduce a single base-pair substitution in two intronic SNPs at the *FTO* locus without altering nearby non-coding sequence. Substitution efficiency increased up to 10-fold by treatment of human embryonic stem cells (ESC) with non-toxic levels of DMSO (1%) before CRISPR/Cas9 delivery. Treatment with DMSO did not result in CRISPR/Cas9 off-target effects or compromise the chromosomal stability of the ESC. Twenty-four hour treatment of human ESC with DMSO before CRISPR/Cas9 delivery may prove a simple means to increase editing efficiency of non-coding DNA without incorporation of undesirable mutations.

## Introduction

The CRISPR/Cas9 system [1–6] is currently the most widely used nuclease-based genome editing tool in human pluripotent stem cells. Compared to zinc-finger nucleases (ZFNs) or transcription activator-like effector nuclease (TALEN), CRISPR/Cas9 offers unparalleled simplicity, specificity, cost effectiveness and overall efficiency of genome editing [7]. For example, efficiencies up to ~ 80% have been reported in introducing random insertions/deletions by a Cas9/guide RNA (gRNA)-mediated double-stranded break (DSB) followed by non-homologous end joining (NHEJ) [8]. Using homology-directed repair (HDR), mutations can be introduced or corrected accurately via CRISPR/Cas9 in combination with single-stranded oligodeoxynucleotide (ssODN) encoding the desired mutation. HDR efficiency is considerably lower than NHEJ [3], but this efficiency can be increased to ~ 80% by introducing a Cas9-blocking mutation targeting the protospacer-adjacent motif (PAM) in the gRNA sequence [9]. When a coding sequence is targeted, the blocking mutation incorporated alongside the pathogenic mutation can be designed so that the amino acid sequence is not changed. In contrast, when non-coding sequence is targeted, the effect of an alteration is less clear and the introduction of a second mutation is undesirable.

**Fig 1:**
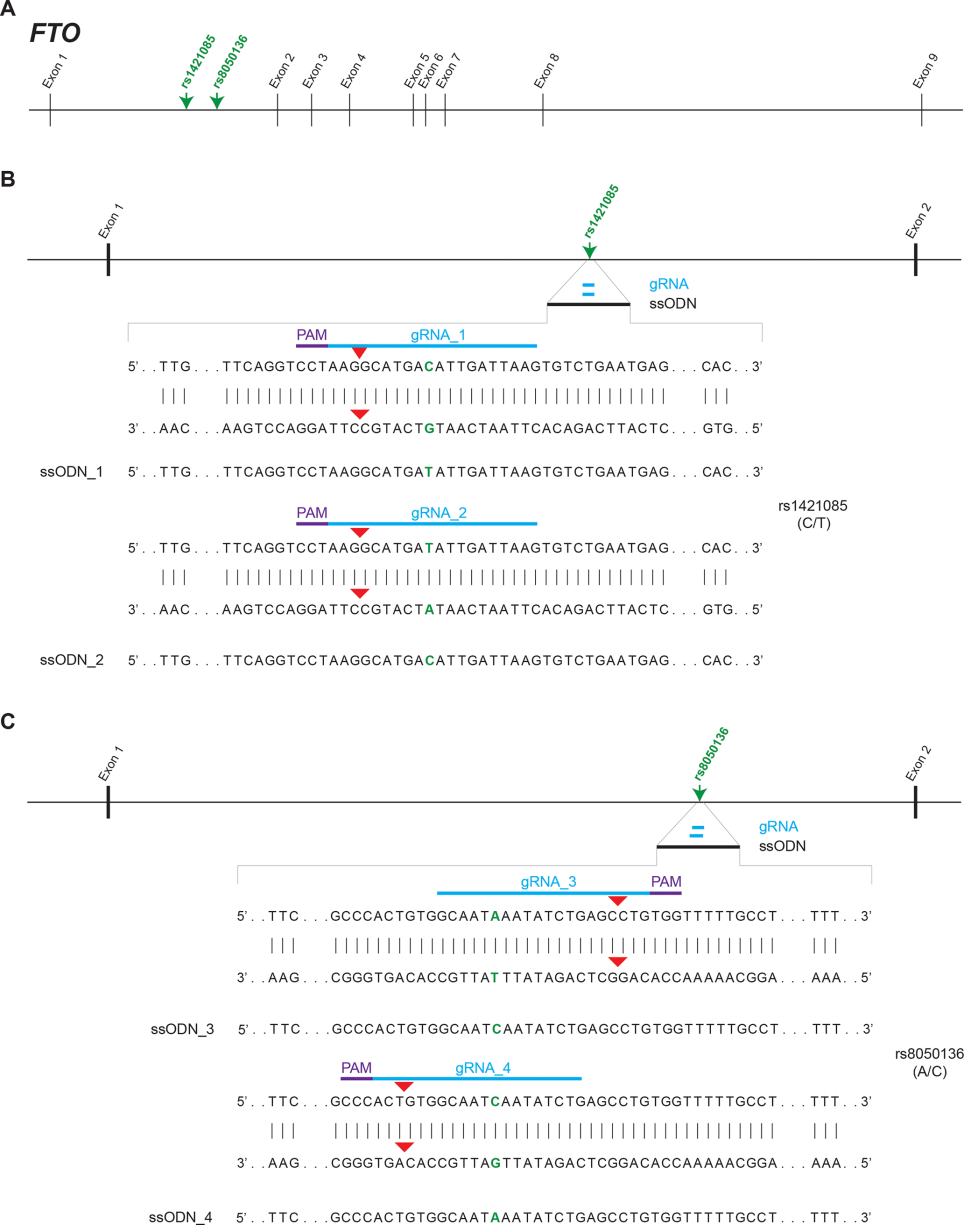
Schematic representation of the *FTO* genomic locus (chr16:53,703,963–54,121,941). (A) SNPs rs1421085 (C/T) and rs8050136 (A/C) are located in the first intron of *FTO*. (B, C) CRISPR/Cas9 technology was employed to convert ESC line H9 (heterozygous for both SNPs) to homozygosity for both alleles at rs1421085 (C/C or T/T) or rs8050136 (C/C or A/A). Positions of gRNA, PAM sequence and ssODN are indicated by thick lines in blue, purple and black, respectively. SNPs are given in green. Predicted Cas9 cut sites are indicated by red arrow heads.

Two single nucleotide polymorphisms (SNPs) in the first intron of the *alpha-ketoglutarate dependent dioxygenase* (*FTO*) gene, rs8050136 and rs1421085 (Fig 1A) have been associated with the regulation of body weight [10–15]. We aimed to convert human ESC line H9, which is heterozygous for both SNPs, to homozygosity for both alleles at rs1421085 (C/C or T/T; C-risk allele) or rs8050136 (C/C or A/A; A-risk allele). Such allelic series may become a valuable tool to study the impact of these variants in stem cell-derived cellular model systems for obesity. Thus, we endeavored to replace a single base at rs8050136 and rs1421085 in human ESC by HDR without introducing a PAM blocking mutation. Our initial attempts to alter rs8050136 and rs1421085 had very low efficiency (0–4%). Here, we report that we achieved an up to 10-fold increase in HDR efficiency by introducing dimethyl sulfoxide (DMSO) in the culture media prior to CRISPR/Cas9 manipulation. This strategy may provide a simple, efficient and cost-effective method for precise and “clean” HDR of non-coding DNA regions otherwise resistant to DNA editing.

## Materials and methods

### Cell lines

The human H9 ESC line was purchased from WiCell. Cells were maintained in a humidified incubator at 37°C on irradiated murine embryonic fibroblasts (MEFs; CF-1 MEF 4M IRR; GLOBALSTEM) in DMEM KO medium (Cat # 10829018; ThermoFisher Scientific) supplemented with 15% KnockOut Serum Replacement (Cat # 10828028; ThermoFisher Scientific), 0.1mM MEM Non-Essential Amino Acids (Cat # 11140050; ThermoFisher Scientific), 2mM GlutaMAX (Cat # 35050061; ThermoFisher Scientific), 0.06 mM 2-Mercaptoethanol (Cat # 21985023; ThermoFisher Scientific), FGF-Basic (AA 1–155), (20 ng/ml media; Cat # PHG0263; ThermoFisher Scientific), 10 mM Rock inhibitor (Cat # S1049; Selleckchem). Cells were passaged using Accutase (Cat # 00-4555-56; ThermoFisher Scientific).

### Plasmid construction

pCas9_GFP was obtained from Addgene (Kiran Musunuru; # 44719). The GFP was replaced by a truncated CD4 gene from the GeneArt® CRISPR Nuclease OFP Vector (ThermoFisher Scientific) by GenScript using CloneEZ® seamless cloning technology resulting in vector pCas9_CD4 (S1 Fig, S1 File).

### CRISPR

**Fig 2:**
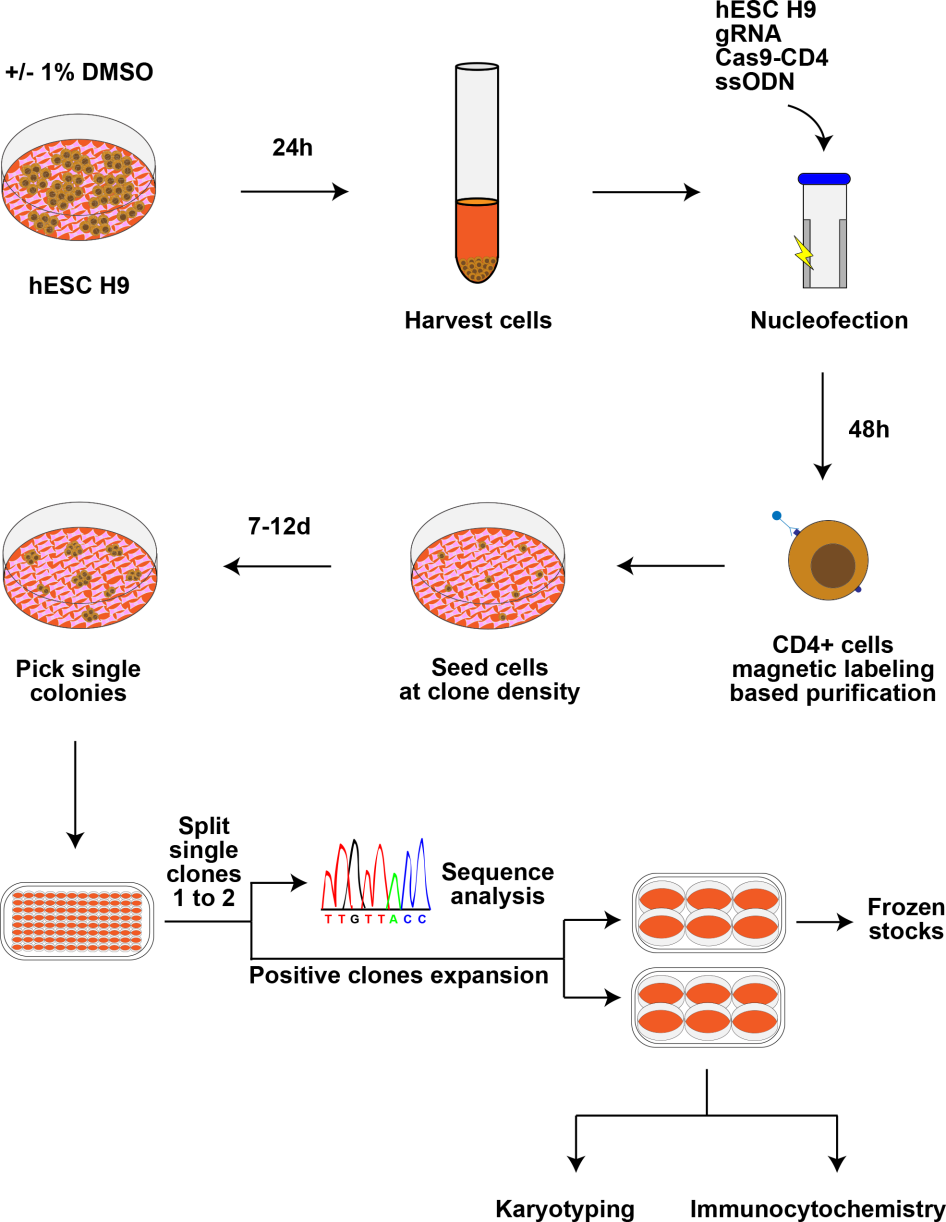
Overview of experimental setup. H9 cells underwent ± DMSO treatment for 24 hours before collection for nucleofection. After 48 hours of recovery after nucleofection, transfected cells were isolated via CD4 bead-based magnetic labeling. They were then seeded at clonal density in 10 cm dishes to facilitate picking of single clones into 96 well plates. After expansion of each clone into two wells of a 96 well plate, genomic DNA was extracted from one well per clone and sequence analysis was performed. Correctly targeted clones were further expanded for freezing, immunocytochemistry, and karyotyping.

The guide RNA sequences gRNA_1: 5’-CTTAATCAATGTCATGCCTT-3’ / gRNA_2: 5’-CTTAATCAATATCATGCCTT-3’ and gRNA_3: 5’-GCAATAAATATCTGAGCCTG-3’ / gRNA_4: 5’-CAGATATTGATTGCCACAGT-3’ utilized to target rs1421085 and rs8050136, respectively. They were designed using Optimized CRISPR Design (MIT; http://crispr.mit.edu/). Cloning of the gRNA into pGS-U6-gRNA was performed by GenScript. The day before nucleofection, 1% v/v DMSO (>99.7% purity; Cat # D2660; Sigma) was added directly to the cell culture media. The following day, 800,000 human ESC were collected and mixed in nucleofection buffer (Cell Nucleofector Kit 2; Cat # VPH-5022) with repair templates (ssODN; IDT, standard desalting) for rs1421085 (0.5 micromolar; 5’- TTGTTCCTCCTGCTACTTAAAATAAAGGTAATATTGATTTTATAGTAGCAGTTCAGGTCCTAAGGCATGAC/TATTGATTAAGTGTCTGATGAGAATTTGTAGGGTAGTCTCCCAGACCTGCAGCTACAGGGCATCTCCCCAC-3’) or rs8050136 (0.5 micromolar; 5’- TTCCCTGGGACCTGTGACAGTGCCAGCTTCATAGCCTAGTCTAGGCATGCCAGTTGCCCACTGTGGCAATA/CAATATCTGAGCCTGTGGTTTTTGCCTTAGGTAAACTGTAGAGATGGACTCATGGAATGCTTGGAAAATTT-3’), gRNA and pCas9_CD4 plasmids (2.5 microgram each). Nucleofection was performed in an Amaxa Nucleofector II (Program A-023) with the Human Stem Cell Nucleofector Kit 2 according to the manufacturer’s instructions. An overview of the experimental approach is given in Fig 2. Cells were plated on MEFs for 2 days for recovery, followed by purification of transfected cells by positive selection of CD4-expressing cells using human CD4 MicroBeads (Cat # 130-045-101; MS Column, Cat # 130-042-20; MACS Miltenyi Biotec) and re-plated at clonal density in 10 cm^2^ tissue culture plates with MEFs. After 7–12 days, ESC colonies were picked into 96-well plates and, 4–5 days later, split 1 : 2 (one well for genomic DNA extraction followed by sequence analysis as described below and one well for amplification of clones and further analysis and freezing if indicated). Each targeting experiment was conducted 3 times for each gRNA.

### Genomic DNA extraction and PCR

Genomic DNA was extracted in wells (96 well plates) by incubation for 1h at 55°C with lysis buffer (20 mM Tris-HCl, 100 mM KCl, 5 mM MgCl2, 0.2 mg/ml Gelatin, 0.9% (v/v) NP-40 and 0.9% (v/v) Tween-20 supplemented with 0.2 mg/mL Proteinase K). Proteinase K was inactivated by incubation at 100°C for 10 min. Rs1421085 (and nearby rs9940128) or rs8050136 (and nearby rs4783819) DNA regions were PCR amplified using Titanium *Taq* DNA Polymerase (Cat # 639211; Clontech) and the following primers: rs1421085 (5’-GGCCTCAGCTTCCCTGAACTG-3’, 5’-GGTTCCCATCTTTAAGGTCAGATTAAGG-3’), rs8050136 (5’- CCTGGGACCTGTGACAGTGCC-3’, 5’-GCCACTCATTCAACCAAAATTCACTACAC-3’). Titanium *Taq* DNA Polymerase performs as a high-fidelity DNA polymerase when amplicons are <1000bp in length, as confirmed by re-amplification with Advantage®2 *Taq* DNA Polymerase (Cat # 639207; Clontech). Sequences were generated by Sanger sequencing (Macrogen). DSBs are defined as the presence of insertions or deletions detected by Sanger sequencing at the DNA site at which each gRNA is predicted to introduce a break.

### Quality controls

The gRNAs specific to rs1421085 or rs8050136 have a score >44 in Optimized CRISPR Design. Two potential off-target sequences with a low score range (4.6–1.3) for each gRNA specific to rs1421085 (gRNA_1 chr6: 5’-CTGAATCAATGTCATGTCTTTGG-3’, chr2: 5’-CTAAAACAATGTCATGCCTTAAG-3’; gRNA_2 chr6: 5’-CTTTATCAATATCATGGCTTCAG-3’, chr5: 5’-CTTAATGAATATCATGCCTAGAG-3’) or rs8050136 (gRNA_3 chr8: 5’-GCTAAAACTATCTGAGCCTGTAG-3’, chr8: 5’-TCTATATATATCTGAGCCTGAAG-3’; gRNA_4 chr6: 5’-CAGTTATATATTGCCACAGTGAG-3’, chr5: 5’-CAGAAATGGAGTGCCACAGTTGG-3’) were assessed by PCR amplification and sequencing using the following primers: rs1421085 gRNA_1 off-target 1: 5’- GGATAAGCACCTGGCACCAAC-3’, 5’-GGAGAATCCCTTGAACTCGAGAGG-3’; rs1421085 gRNA_1 off-target 2: 5’-CATTCTTCACTCCTTTCTTAATGACATTACCTAG-3’, 5’- CCGTGGTTTCATGTTGTTATGGCC-3’; rs1421085 gRNA_2 off-target 1: 5’-CCTACATGGTGTGTTATCTCCTTAAAGG-3’, 5’-CATACAAAGGGCCTTCTACTCTTTTGC-3’; rs1421085 gRNA_2 off-target 2: 5’-GACGTTGACTGTATCAGTTACCTTTC-3’, 5’-CAAATGCCAGTGCTATTGCAGGTC-3’; rs8050137 gRNA_3 off-target 1: 5’- GTATCTCCTTTGTACTGTTGTTGAAAACC, 5’-GTGTATCAGCATTAAGTAAGAACACCGTG-3’; rs8050137 gRNA_3 off-target 2: 5’-GTACTTTCATGTTTTCATTTCTTGCATTTAGATCCACATG-3’, 5’-TTGGGAGGCTGAGGTGGGAGG-3’; rs8050137 gRNA_4 off-target 1: 5’- GAGCAGACAGGGCTGAGTTG-3’, 5’-GGCAAACATAGAATTGAGCTGGTGG-3’; rs8050137 gRNA_4 off-target 2: 5’-CTGAACCAAGAACACTGCGCTG-3’, 5’- GGGGAAAGCAAAAGCAGAGATCTGATTG-3’. The remainder of the potential off-target sequences (~200 per gRNA) are highly improbable targets (scores <1).

### Karyotyping

G-band karyotyping analysis was done by Cell Line Genetics. Chromosome analysis was performed on 20 cells per cell line.

### Immunofluorescence

ESC clones were fixed on culture slides in 4% paraformaldehyde for 10 min followed by cell permeabilization with 0.1% Triton X. Cells were stained for pluripotency markers SOX2 (1:400; Cat # 09–0024; Stemgent), OCT4 (1:200; Cat # sc-5279; Santa Cruz Biotechnology), TRA-1-81 (1:200; Cat # mab8495; R&D Systems), SSEA-4 (1:200; Cat # MAB1435) and NANOG (1/100; Cat # AF1997; R&D SYSTEMS).

### Statistical analysis

Data are expressed as means ± standard deviation. Statistical analysis was performed using Student's T-test (StatView 5.0, SAS Institute Inc.). Levels of statistical significance were set at 2-tailed *p*_alpha_<0.05.

## Results

**Fig 3:**
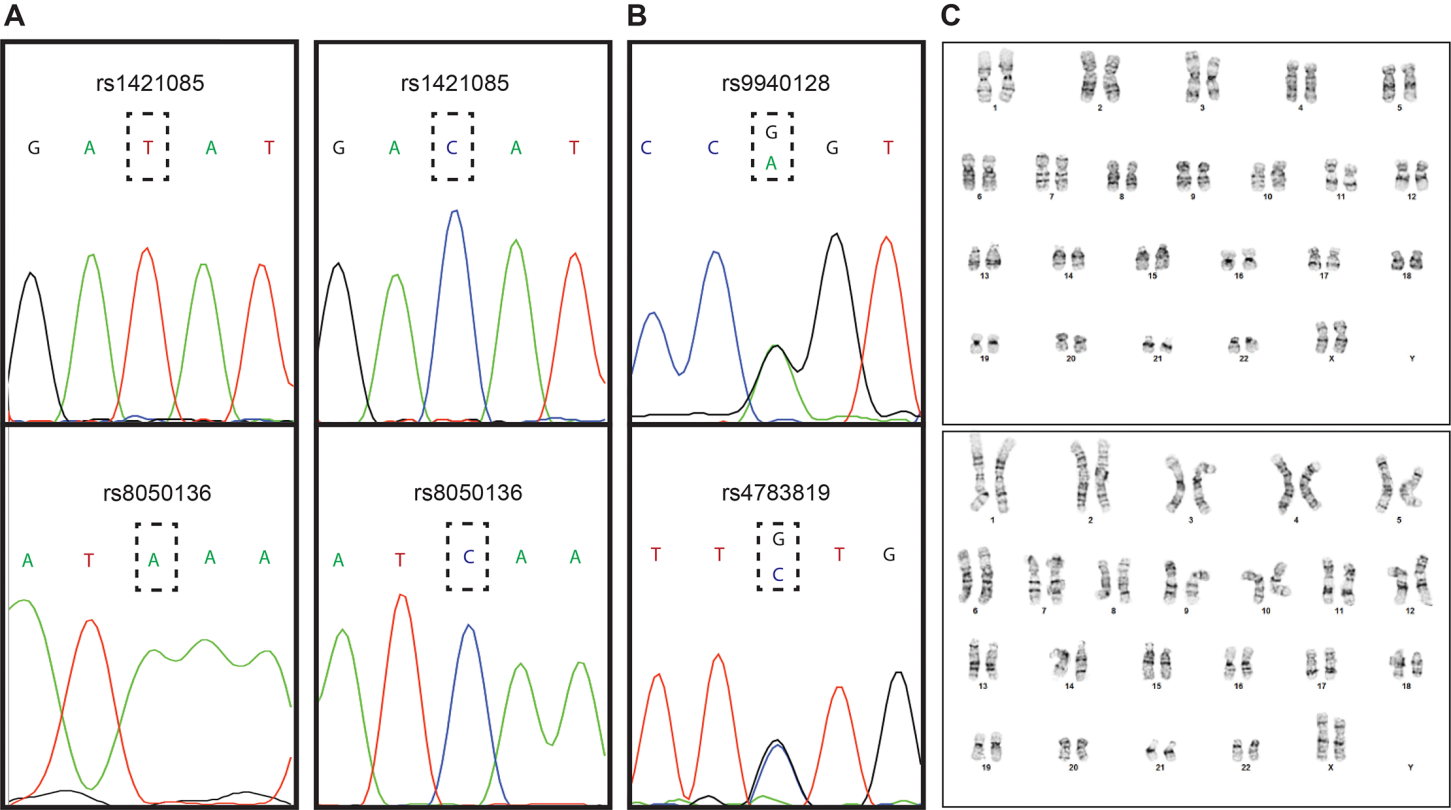
Quality control measures of correctly targeted clones. (A, B) Results from Sanger sequencing. The SNPs rs9940128 (near rs1421085) and rs4783819 (near rs8050136) were amplified and sequenced in the same read to control for possible long deletions in the correctly targeted ESC clones. (C) Two representative karyotypic images. All correctly targeted clones tested displayed a normal karyotype.

**Fig 4:**
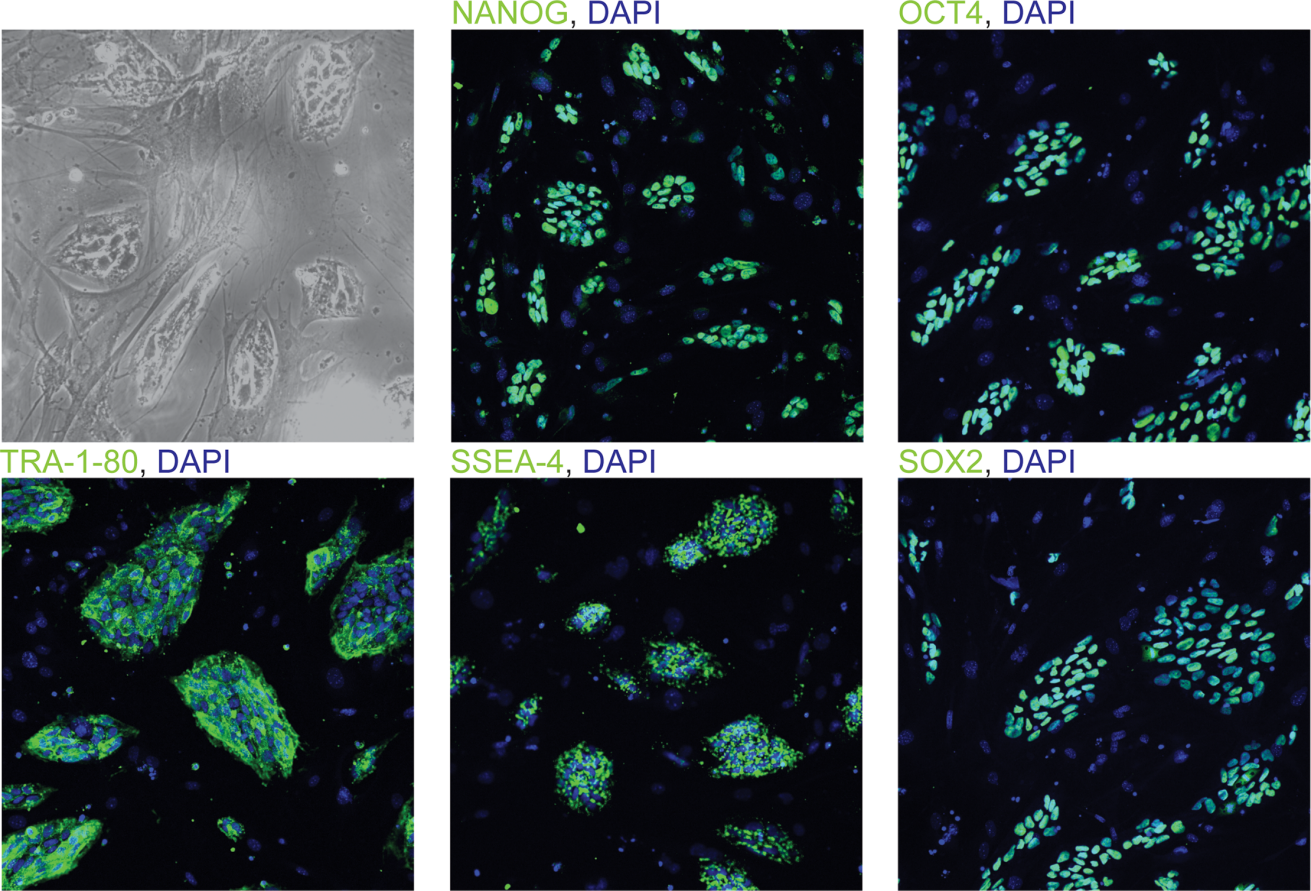
Correctly targeted ESC clones are pluripotent. Immunofluorescence of correctly targeted ESC clone rs1421085 (C/C) showing pluripotency molecular markers homeobox transcription factor NANOG, octamer-binding homeodomain transcription factor 4 (OCT4), glycoprotein TRA-1-80, Stage-Specific Embryonic Antigen-4 (SSEA-4), and transcription factor SRY (sex determining region Y)-box 2 (SOX2). Pluripotency markers are shown in green, nuclei are counterstained with DAPI.

The human H9 ESC line is heterozygous for SNPs rs8050136 (A/C) and rs1421085 (C/T) within the first intron of *FTO*. H9 ESC cells were treated with 1% DMSO [16] 24 h before nucleofection with plasmid pCas9_CD4 (S1 Fig, S1 File) carrying both Cas9 and a truncated CD4 that enables bead-based enrichment of *Cas9*-expressing cells. The nucleofection mix also included a plasmid with the A/C-allele-specific (rs8050136), or T/C-allele-specific (rs1421085) gRNA sequence driven by the U6 promoter (see Methods), and a ssODN complimentary to C/A at rs8050136, or C/T at rs1421085. After CD4 bead-based enrichment, 100 colonies were picked from each “CRISPRed” cell pool that had been treated with DMSO and 100 colonies from the “CRISPRed” untreated cell pool. By Sanger sequencing, we determined that 4 to 10-fold more clones were successfully targeted in DMSO-treated cells than in non-treated controls (Table 1). DMSO also resulted in a ~ 30% increase of DSBs targeting A at rs8050136, and a ~ 2-fold increase of DSBs targeting C at rs8050136 or C at rs1421085 (Table 1). We assessed the sequence of SNPs near rs8050136 and rs1421085 in order to verify that the correctly targeted clones did not contain long deletions at rs8050136 and rs1421085 (Fig 3). Furthermore, we assessed chromosomal stability by karyotyping and the correctly targeted clones showed a normal karyotype (Fig 3). We assessed potential off-target effects by sequencing each clone for the two gRNA genomic targets with the highest score in Optimized CRISPR Design (Methods) and did not detect any variants. Finally, we confirmed the pluripotency status of the correctly altered clones (Fig 4).

**Table 1:** Percentage of HDR, DSB and no cleavage in DMSO-treated and non-treated clones.

	HDR		*P value*	DSB		*P value*	No cleavage		*P* *value*
	DMSO (%)	- (%)		DMSO (%)	- (%)		DMSO (%)	- (%)	
rs8050136 (C/C)	23 ± 5	3 ± 1	0.002	66 ± 4	44 ± 9	0.02	11 ± 4	53 ± 8	0.001
rs8050136 (A/A)	17 ± 2	4 ± 1	<0.001	71 ± 3	40 ± 2	<0.001	12 ± 5	56 ± 2	<0.001
rs1421085 (C/C)	22 ± 4	2 ± 1	0.001	44 ± 6	35 ± 5	0.1	34 ± 6	63 ± 6	0.003
rs1421085 (T/T)	11 ± 2	1 ± 1	<0.001	54 ± 3	23 ± 7	0.002	35 ± 5	76 ± 8	0.001

Abbreviations

HDR—Homology Directed Repair; DSB–Double Stranded Break

## Discussion

Many efforts have been made to improve CRISPR/Cas9 editing efficiency at various genomic loci [17, 18]. Critical parameters affecting efficiency include dependence on cell cycle stages amenable for HDR [19], the DNA accessibility of the target locus [20] and the efficiency of sgRNA assembly into Cas9 [4]. DMSO has been reported to induce reversible G1 arrest [21], which can increase the capacity of human pluripotent stem cells to differentiate [16, 22]. However, this effect of DMSO is probably not the explanation for the 6 to 10-fold increase in HDR efficiency reported here. Borowiak and colleagues demonstrated in an earlier study that enrichment of pluripotent stem cells in G2/M enhances HDR-mediated gene repair [19].

A genome-wide Cas9 binding study (chromatin immunoprecipitation followed by sequencing) in mouse embryonic stem cells observed that chromatin-mediated DNA inaccessibility decreases Cas9 binding [20]. For many years, DMSO has been used to resolve DNA secondary structure and DNA supercoiling in PCR reactions [23, 24]. DMSO has been shown to alter the expression levels of DNA methylation enzymes with effects on DNA methylation as well as hydroxymethylation [25, 26]. It is conceivable that DMSO may facilitate DNA access for the CRISPR/Cas9 enzyme. Importantly, we show that two potential off-target loci were not altered by the inclusion of DMSO in the cell incubates, and that no gross karyotypic alterations were detected. However, considering the increased DNA access as the potential mechanism for the improved HDR efficiency in the DMSO condition, careful off-target analysis and karyotyping in DMSO-treated cells is a prerequisite for utilization of this tool.

Doudna and colleagues showed that, upon expression in human cells, Cas9 localizes to the nucleus and assembles with sgRNA *in vivo*. The sgRNA assembly into Cas9 is the limiting factor for Cas9-mediated DNA cleavage [4], suggesting that the Cas9/sgRNA ratio and/or delivery method could potentially impact the efficiency of cleavage. Various delivery systems have been used to facilitate sufficient expression of Cas9 and gRNA in mammalian cells, e.g: Lipofectamine 2000 [27], cell-penetrating peptides [28], lipid-like nanoparticles [29], purified recombinant Cas9 and guide RNA [30], lentiviral [31–33] and adeno-associated (AAV) virus [34]. Lentiviral transduction relies on random integration into the genome, AAV-delivered gRNA/Cas9 can be associated with persistence of gene expression, and purified gRNA/Cas9 may not be cost-effective.

In this study we have shown that brief treatment with DMSO is potentially a simple and cost-effective method to increase efficiency of the CRISPR/Cas9 system. We believe that nucleofection and transient expression of gRNA, Cas9, and CD4 provides an efficient method of delivery and enrichment for transfected cells. This strategy may be an alternative for manipulation of non-coding loci which cannot be targeted using routine high efficiency methods such as ssODN carrying a silent mutation in the PAM. An alternative or synergistic strategy to improve the efficiency and on-target cleavage is the optimization of the sgRNA structure [35]. Accumulating knowledge of the detailed molecular mechanisms underlying the CRISPR/Cas-induced DNA cleavage will enable development of improved strategies to efficiently target genomic loci and reliably induce HDR, resulting in successful genome editing [35].

## Supporting information

S1 Fig pCas9_CD4 plasmid map. This plasmid facilitates the co-expression of human codon-optimized Cas9 and a truncated version of CD4. The vector carries the insert Cas9-2A-CD4 under the control of the pCAG (CMV enhancer/chicken β-actin) promoter.
(TIF)

S1 File pCas9_CD4. Nucleotide sequence of pCas9_CD4. Human codon-optimized Cas9 is underlined and CD4 is given in bold. The vector backbone is that of pCas9_GFP (Addgene # 44719).
(DOCX)
