# Supplementary figures and images for: DMSO increases efficiency of genome editing at two non-coding loci
Source: PLoS One. 2018 Jun 4;13(6):e0198637. doi: 10.1371/journal.pone.0198637 (PMC5986138; doi:10.1371/journal.pone.0198637)

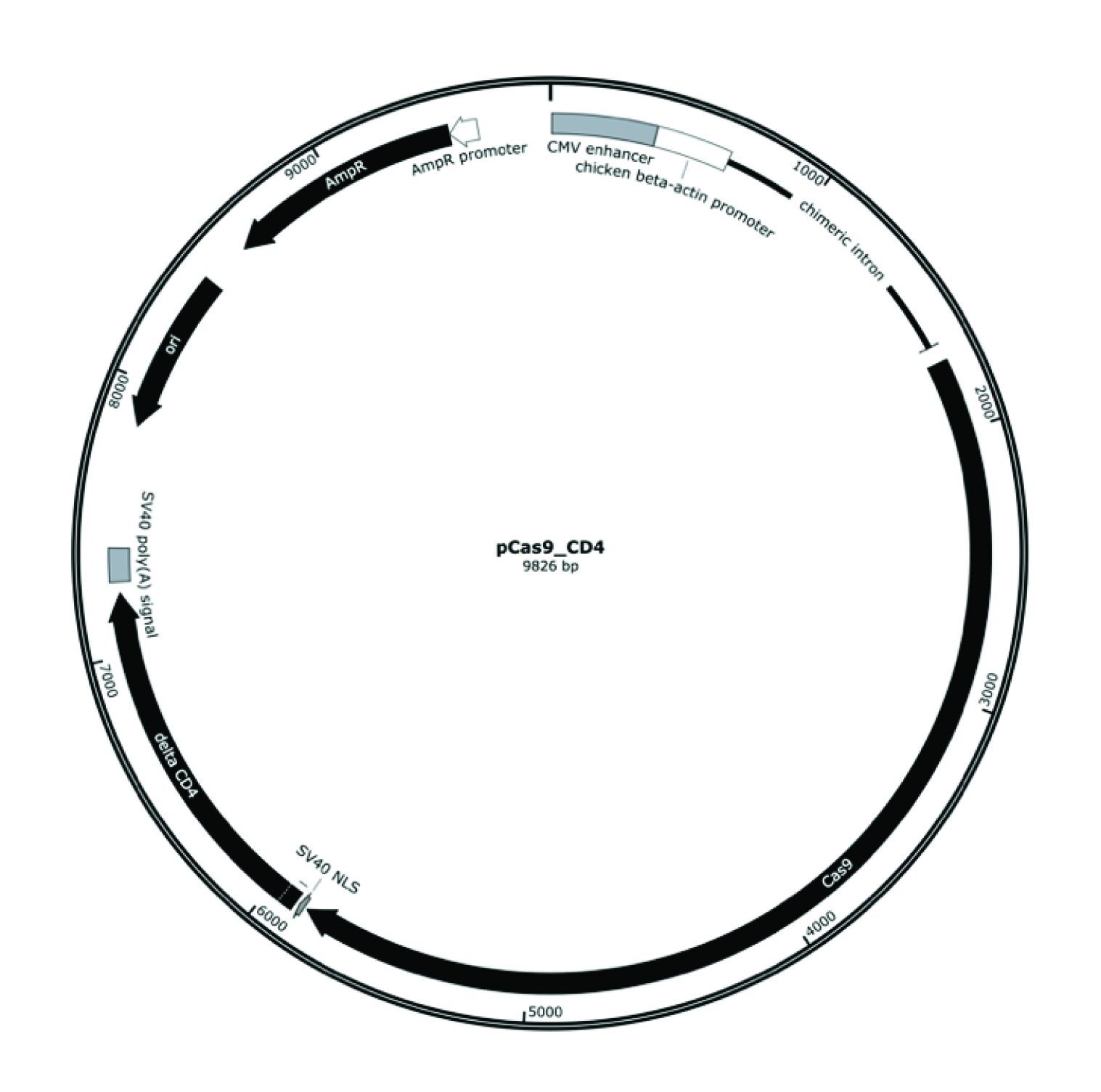

Supplement: S1 Fig — This plasmid facilitates the co-expression of human codon-optimized Cas9 and a truncated version of CD4. The vector carries the insert Cas9-2A-CD4 under the control of the pCAG (CMV enhancer/chicken β-actin) promoter. (TIF) [file pone.0198637.s001.tif]
